# Objectively Measured Physical Activity and Fat Mass in Children: A Bias-Adjusted Meta-Analysis of Prospective Studies

**DOI:** 10.1371/journal.pone.0017205

**Published:** 2011-02-23

**Authors:** Desiree C. Wilks, Stephen J. Sharp, Ulf Ekelund, Simon G. Thompson, Adrian P. Mander, Rebecca M. Turner, Susan A. Jebb, Anna Karin Lindroos

**Affiliations:** 1 Medical Research Council Collaborative Centre for Human Nutrition Research, Cambridge, United Kingdom; 2 Department of Prevention and Sports Medicine, Technische Universität München, Germany; 3 Medical Research Council Epidemiology Unit, Cambridge, United Kingdom; 4 Medical Research Council Biostatistics Unit, Cambridge, United Kingdom; University of Swansea, United Kingdom

## Abstract

**Background:**

Studies investigating the prevention of weight gain differ considerably in design and quality, which impedes pooling them in conventional meta-analyses, the basis for evidence-based policy making. This study is aimed at quantifying the prospective association between measured physical activity and fat mass in children, using a meta-analysis method that allows inclusion of heterogeneous studies by adjusting for differences through eliciting and incorporating expert opinion.

**Methods:**

Studies on prevention of weight gain using objectively measured exposure and outcome were eligible; they were adopted from a recently published systematic review. Differences in study quality and design were considered as internal and external biases and captured in checklists. Study results were converted to correlation coefficients and biases were considered either additive or proportional on this scale. The extent and uncertainty of biases in each study were elicited in a formal process by six quantitatively-trained assessors and five subject-matter specialists. Biases for each study were combined across assessors using median pooling. Results were combined across studies by random-effects meta-analysis.

**Results:**

The combined correlation of the unadjusted results from the six studies was −0.04 (95%CI: −0.22, 0.14) with considerable heterogeneity (I^2^ = 78%), which makes it difficult to interpret the result. After bias-adjustment the pooled correlation was −0.01 (95%CI: −0.18, 0.16) with apparent study compatibility (I^2^ = 0%).

**Conclusion:**

By using this method the prospective association between physical activity and fat mass could be quantitatively synthesized; the result suggests no association. Objectively measured physical activity may not be the key determinant of unhealthy weight gain in children.

## Introduction

The prevalence of obesity in childhood has increased around the world [1], [2]. This is a great concern as childhood obesity is associated with many immediate and long-term health consequences [3]–[6]. The fundamental physiological cause of accumulation of excess fat mass is a positive energy imbalance due to higher energy intake than expenditure. Accordingly, it has been suggested that an increase in energy expenditure due to more physical activity (PA) is important in maintaining energy balance and protecting against excess weight gain [6]. There is compelling evidence for a strong inverse cross-sectional relationship between PA and body weight, fat mass and obesity [7], [8]. However, concerns of reverse causality hamper the interpretation of these results, that is higher body weight may lead to a lower PA level rather than vice versa. Moreover, the evidence from prospective studies of an association between PA and measures of adiposity is less clear [9]. Conflicting results may partially be due to many studies using self-reported methods such as questionnaires or recall interviews to assess PA [10], which are susceptible to misclassification and recall bias [11]. Recently, there has been an increase in the number of studies using objective methods to assess PA and physical activity related energy expenditure, such as the doubly-labeled water method or accelerometers. These methods estimate PA and physical activity energy expenditure with high precision and accuracy [12]–[16], which is important in fully understanding the effects of PA on body weight and other health outcomes.

Conflicting results in studies of PA and adiposity may also be caused by different aspects of study design and quality such as varying duration of follow-up, study populations, or analysis methods. These differences make it difficult to combine studies and obtain an overall pooled estimate of the association, since the results from a conventional meta-analysis reflect only uncertainty due to random variation and do not acknowledge systematic biases [17], [18]. A common approach to handling variation in design and quality is to exclude studies with certain characteristics, but this introduces an artificial division among available studies and valuable evidence may be lost [19]. To overcome this problem and to be able to estimate an overall pooled estimate of the association, we have adapted and applied a recently developed meta-analysis method that allows adjustment for differences in study design and quality through a formal process of eliciting and incorporating expert opinion [20]. This method attempts to quantify the biases and their uncertainty, independently of the results, rather than to ignore them and produce a pooled association, which is difficult to interpret. Although the use of expert opinion may be considered controversial, meta-analysts routinely rely on even stronger judgments when excluding some studies altogether and regarding those included as unbiased. Moreover, policy makers faced with imperfect evidence use expert opinion informally in making judgments and decisions. The aim of this research was to formalize this process, making it transparent and accountable, and use this novel meta-analysis method to quantitatively synthesize the evidence on the prospective association between measured PA energy expenditure and change in percent body fat in children in order to better inform evidence based policy making with respect to PA strategies.

## Materials and Methods

### Source studies

This meta-analysis includes all prospective observational studies investigating the association between total daily PA and subsequent change in adiposity (n = 6) [21]–[26] presented in a previous systematic review, which considered reports published between January 2000 and September 2008 [27]. Inclusion criteria of the review were that both body composition and PA were assessed objectively. PA was assessed by doubly-labeled water, indirect calorimetry, heart rate monitors or accelerometry. Doubly-labeled water and heart rate methods estimate the total energy expenditure, which can then be used to calculate PA energy expenditure [28]. Accelerometers measure total body acceleration and provide information on total body movement, the total amount of time spent in PA and the PA intensity [29]. The outcome of interest was a body fat measure, assessed objectively for example by dual-energy X-ray absorptiomety (DXA) or skinfold callipers. Studies were excluded if their samples were either limited to clinically ill participants, or if they originated from trials involving intentional weight loss.

### Application of the bias-adjustment method

The bias-adjustment method is described in detail by Turner et al and Thompson et al [20], [30]. The steps used to implement this method for the six studies included in this meta-analysis are outlined below. For clarification of the method, the study by Johnson et al [23] is used as an example throughout this paper.

### Target question and target setting

A precise definition of the public health target question, which the meta-analysis aims to address, was agreed as: “Is physical activity associated with subsequent change in fat mass in children?”.

The target setting, which describes a potential study protocol to answer the target question with regards to study population, exposure and outcome measures and follow-up time, was defined as:

- General population of children aged 4–11 years in the UK.
- PA energy expenditure measured at baseline.
- Subsequent change in percent body fat, measured at baseline and follow-up.
- Outcome assessed two years after the baseline measurement.

The target setting focuses on children between four and 11 years excluding both baby to toddler stages and advanced stages of puberty to match the policy focus on the UK Healthy Weight Healthy Lives strategy [31]. PA energy expenditure was used as the target exposure due to its contribution to energy balance. Percent body fat was the best measurement of adiposity in children that was reported in the eligible studies. A two-year follow-up was selected since we anticipated diminished associations with increasing follow-up time, whereas shorter follow-up times would not adequately allow for the slow accumulation of fat mass.

### Idealized studies

For each study included in the meta-analysis, an idealized version was defined. The idealized study is a theoretical repeat of the actual study with modifications to eliminate all sources of internal biases such as selection bias, attrition bias, inappropriate adjustment for confounding and biases arising from how the exposure and outcome were measured. The design of the idealized study does not need to be practically feasible. For example, the idealized version of the study by Johnson et al [23] included the following elements:

- 4–11 year old girls and boys, who are free of any major illness since birth from Birmingham in Alabama, USA.
- PA energy expenditure measured at baseline.
- Rate of increasing adiposity (kg fat/kg lean) measured at baseline and follow-up.
- Outcome assessed four years after the baseline measurement.

### Internal and external biases

Potential internal biases in each study were identified by comparing the study against its idealized version. For this meta-analysis, internal biases were categorized as biases related to the measurement of the outcome (“outcome bias”) and the exposure (“exposure bias”), missing data and loss to follow-up (“attrition bias”), whether the confounders in the analysis were appropriate (“confounding bias”) and whether the inclusion and exclusion criteria were clear and adhered to (“selection bias”). Biases related to inappropriate statistical analysis or any other flaws were included in a separate category (“other bias suspected”). Important variables potentially related to both the outcome and exposure were considered by the subject-matter specialists and statisticians and the following reference set of confounders was selected: baseline fat free mass and fat mass, Tanner stage or age, ethnicity and sex. The adjustment for confounding used in each study was judged against this reference set.

Potential external biases were identified by comparing each idealized study against the target setting. External biases were categorized as biases related to the follow-up time (“timing bias”), the presented outcome (“outcome bias”) and exposure measures (“exposure bias”) and the study population (“population bias”).

Bias checklists were prepared for each study, highlighting information that might be relevant in the assessment of each of the possible internal and external biases. To ensure consistency, biases were identified by the same subject-matter specialist for all studies together with one statistician for each study.

### Common scale for study results

The studies expressed the association between PA and change in adiposity differently, with a mix of regression coefficients, *P*-values and R-squared values. To allow the results to be pooled it was necessary to transform the associations onto a common metric. All studies reported a *P*-value and the sample size, which enabled calculation of a correlation coefficient and standard error for each study. Biases were assessed on the correlation scale. To perform the calculations, the Fisher transformation of the correlation was used: *z* = 0.5 ln[(1 + *r*)/(1 - *r*)] and *z* having a standard error SE(*z*)  = 1/√(*n* - 3), where *r* is the correlation and *n* is the sample size. The number of SEs *z* is away from zero is derived from the *P*-value, thus providing an estimate of *z*. We back-transformed *z* to the correlation *r* in order to present results; these scales are in fact almost identical in the range −0.3 to +0.3. Data were analyzed using STATA 11.0 (StataCorp 2009. College Station, TX: StataCorp LP).

### Bias elicitation meetings

**Figure 1:**
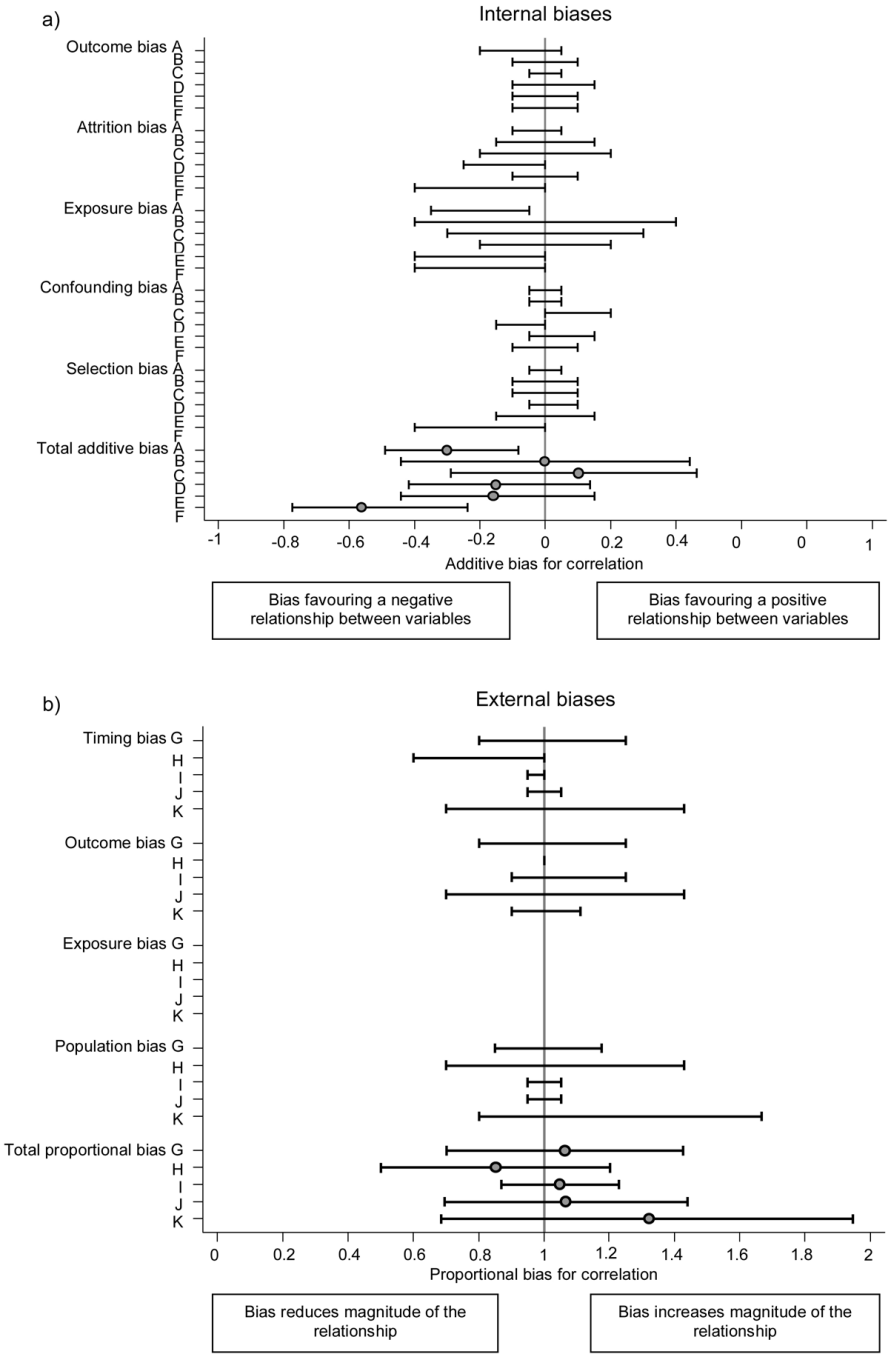
Assessment of biases for the study by Johnson et al (23). In this study all internal biases were additive and all external biases were proportional. Internal biases were elicited from six assessors (A–F) and external biases from five assessors (G–K). Ranges indicate 67% confidence intervals for the bias, so the bias is considered twice as likely to be inside the interval as outside it. A blank indicates no bias for that category.

Internal bias elicitation meetings involved six quantitatively-trained assessors and separate external bias elicitation meetings involved five assessors with subject-matter knowledge. At the meetings, each study report and bias checklist were discussed in turn in a non-quantitative manner; the discussion included consideration of whether each bias would only change the magnitude of the association (a proportional bias) or if it could change the direction of the association (an additive bias). Following the discussion, each assessor independently provided their opinion on the impact and uncertainty of each of the biases on a bias elicitation form. The biases were indicated using 67% confidence intervals, such that the assessor thought that the bias was twice as likely to lie inside the interval as outside it. The additive and proportional elicitation scales for quantifying internal and external biases are shown in Figure 1 (x-axes). For example if the assessor believed that losses to follow-up introduced a small additive attrition bias but was not able to anticipate the direction of the bias, a possible 67% interval could be (−0.1, 0.1). If the assessor believed a study with 18 months follow-up instead of two years introduced a small proportional timescale bias then a possible 67% interval could be (0.9, 1).

### Incorporating the bias elicitations into the meta-analysis

The elicited internal biases from each assessor were used to calculate the mean and variance of the total additive and total proportional bias for each study, which were then used to adjust the estimated correlation coefficients and standard errors. The same process was then used to adjust these results for the external biases. All calculations used formulae adapted from Turner et al [20]. The results were pooled across assessors, using the median estimate and the median standard deviation; such median pooling corresponds to a “typical” assessor [32]. Finally, the fully adjusted results were combined across studies using random-effects meta-analysis. Statistical heterogeneity was assessed using the I^2^ statistic [33], which gives the percentage of variation between the study estimates attributable to true between-study heterogeneity rather than random variation; 0% indicates no heterogeneity.

## Results

### Study characteristics and extracted results

Table 1 summarizes the six eligible prospective studies on measured PA and change in adiposity. Studies were all carried out in the USA, four of them involving children of mixed ethnicity [21]–[23], [26], one involving Caucasians [24] and one involving Pima Indians [25]. Two studies examined girls only [22], [26], whilst all other study populations consisted of both boys and girls aged between three and 11 years. The studies included between 39 and 138 children in their prospective analysis, with three studies including >100 children [21], [23], [25]. Reported drop-out rates were either 15% or less [21], [23]–[26] or approximately 20% [22]. Follow-up time varied between one and eight years.

**Table 1:** Summary of study characteristics.

Study	Population	Follow-up	Exposure	Outcome^3^	Confounding adjusted for in the models
DeLany et al [21]	131 9 to 11 year old healthy, lean and obese African-American and Caucasian children from Baton Rouge, USA.	2 years	TEE^1^	Δ%BF	No confounders used in the principal results extracted.
Figueroa-Colon et al [22]	47 5 to 9 year old healthy, normal weight Caucasian, African-American or Asian-American girls from Birmingham, USA.	1.6 years	PAEE^2^	ΔBF	*BL* FFM and BF
Johnson et al [23]	115 4 to 11 year old healthy African-American and Caucasian children from Birmingham, USA.	3 to 5 years	PAEE^1^	FM/FFM	Ethnicity, *BL* FM, FFM, Tanner stage and age. Sex according to the abstract.
					
Moore et al [24]	103 3 to 5 year old healthy Caucasian children, whose parents are 3^rd^ or 4^th^ generation of the Framingham Heart Study, USA.	Annual *FU* for 8 years	Average accelerometer counts over 8y	SSF	Sex, *FU* age and *BL* BMI
					
Salbe et al [25]	138 5 year old healthy Pima Indian children from Arizona, USA.	5 years	PAEE^1^	*FU* %BF	*BL* %BF and sex
Treuth et al [26]	101 8 to 9 year old healthy lean African-American and Caucasian girls in Tanner stage 1 living in Houston, USA.	2 years	PAEE^1^	Δ%BF	PAEE, group (according to parental obesity), *BL* time, ethnicity, *BL* Tanner stage, *BL* %BF, group and *BL* time interaction.

BF  =  body fat; *BL*  =  baseline; Δ  =  change; FFM  =  fat free mass; FM  =  fat mass; *FU*  =  follow-up; SSF  =  sum of skinfolds; DXA  =  dual-energy X-ray absorptiometry assessment; PAEE  =  physical activity energy expenditure; TEE  =  total daily energy expenditure.

1 EE measured by doubly-labeled water.

2 EE measured by whole room indirect calorimetry.

3 Outcome measured by DXA except for Moore et al (skinfold caliper) and Salbe et al (DXA & ^18^O).

The exposure was a measure of energy expenditure in all studies except for one, which used accelerometer counts [24]. Total energy expenditure was assessed by either doubly-labeled water [21], [23], [25], [26] or by 24 hour whole room indirect calorimetry [22]. Resting energy expenditure was assessed either by indirect calorimetry [22], [25] or estimated from published equations [21], [23], [26]. In four studies PA energy expenditure was reported and used as the exposure in the meta-analysis [22], [23], [25], [26]; one study only reported results of interest on total energy expenditure, which was therefore used [21]. Body composition was measured by DXA [21]-[23], [26], both DXA and ^18^O [25] or skinfold calipers [24]. The reported outcome was change in percent body fat in four studies [21], [22], [25], [26], while one [23] used the ratio of fat mass and lean total mass. One study presents the association of average accelerometer counts assessed annually over the ages 4–11 years (categorized into activity tertiles) and the sum of skinfolds at 11 years [24]. With the exception of one study [21], the estimated association between PA and change in adiposity was adjusted for confounding factors. Depending on the study, these included a baseline measure of body composition, sex and ethnicity.

Table 2 presents the results extracted from each study along with calculated correlation coefficients. Three studies reported inverse associations between either baseline energy expenditure and change in percent body fat (p<0.05) [21], [22] or accelerometer counts and the sum of skinfolds (p-value for trend  = 0.045) [24]. One study reported a positive association between baseline PA energy expenditure and change in percent body fat [25], whilst two other studies found no significant associations (*P* = 0.74 and *P* = 0.14) [23], [26].

**Table 2:** Correlation coefficients of studies calculated from *P*-values according to the principal results extracted.

Study	Extracted results		Re-calculated results	
(source for extracted result)	n	*P*-value	r (SE)	z (SE)^1^
DeLany et al [21] 2	114	0.04	−0.19 (0.09)	−0.19 (0.09)
Figueroa-Colon et al [22] 3	39	0.04	−0.33 (0.16)	−0.34 (0.17)
Johnson et al [23] 4	113	0.74	0.00 (0.09)	0.00 (0.10)
Moore et al [24]	94	0.045	−0.21 (0.10)	−0.21 (0.10)
Salbe et al [25] 5	138	0.003	0.25 (0.09)	0.26 (0.09)
Treuth et al [26] 6	88	0.14	0.16 (0.11)	0.16 (0.11)

1 Fisher-transformed correlation.

2 The reported value is *P*<0.04.

3 The reported *P-*value (0.04) is for PAEE only, not for the whole model.

4 The correlation giving *P* = 0.74 is +0.03 or −0.03 (*P*-value for trend). We use calculated r = 0.00 as an approximation of these two values.

5 The analysis presented in the source study directly addresses PAEE and is not a stepwise regression.

6 This repeated measures ANOVA uses both year 2 – year 1 changes and year 1 – year 0 changes. The sample size is 88 subjects, but the effective sample size is somewhere between 88 and 2 * 88 = 176 depending on the correlation between individual changes (respectively from 1 to 0).

### Bias-adjusted meta-analysis

Table S1 summarizes the internal biases of the six source studies. An internal bias suspected to affect five source studies was selection bias [22]–[26]. It was judged that insufficient information had been provided in the source and related papers regarding the recruitment strategy, the non-participation rate or immediate drop-outs. In five studies attrition may have affected the results [21]–[24], [26]. Confounding bias was suspected in all studies, mainly because not all relevant confounders were adjusted for or the choice of confounders was not justified. Two studies were considered to have internal outcome and exposure biases, because of the way the exposure or the outcome measures were used in the analysis [23], [24]. Four studies presented a statistical analysis that may have introduced bias [21], [22], [24], [26].

External biases were expected due to differences between the source studies and the pre-defined target setting regarding the population, follow-up time and the outcome and exposure measures (Table 1).

Figure 1 shows the individual assessments of each bias assessor on the elicitation scales for internal and external biases in the study by Johnson et al [23]. In this study all internal biases were considered additive and all external biases proportional. Internal biases were generally distributed around −0.15, suggesting that the biases are believed to be likely to induce an association between exposure and outcome which is more negative than the truth, although the uncertainty was large. The proportional external biases tended to be distributed around one, that is the biases were considered equally likely to favor an exaggeration or an attenuation of the association. The uncertainty was also high for the external biases. Despite the fact that biases were elicited independently, there was a general degree of consistency amongst assessors.

**Figure 2:**
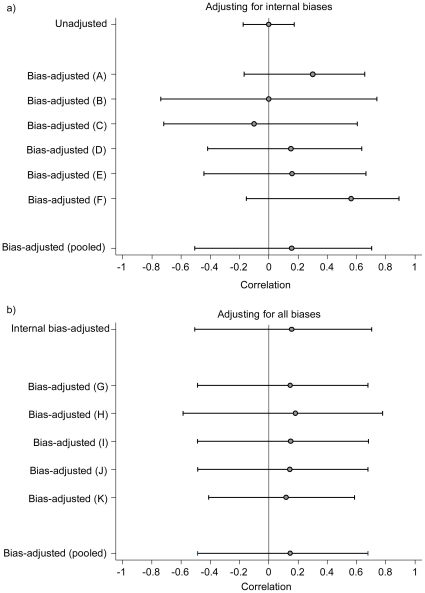
Adjusting for bias for the study by Johnson et al (23). Shown is the impact on correlations (95% confidence intervals) of adjusting for bias for the assessors (A–F and G–K) separately and combined using median pooling. Values on the left hand side of the x-axis represent a negative correlation between physical activity and change in adiposity, i.e. greater baseline physical activity is related to a smaller increase in adiposity.

The impact of adjusting the estimated correlation from the study by Johnson et al [23] for first internal and then external biases is illustrated in Figure 2. Since most assessors judged that the internal biases favored a negative association between PA and change in adiposity, the bias-adjusted correlation was shifted in a positive direction. When the external biases were also incorporated, the point estimate of the correlation hardly changed. However, the considerable uncertainty in the size of both internal and external biases meant the 95% confidence interval for the bias-adjusted estimates was substantially wider than the confidence interval around the unadjusted result.

**Figure 3:**
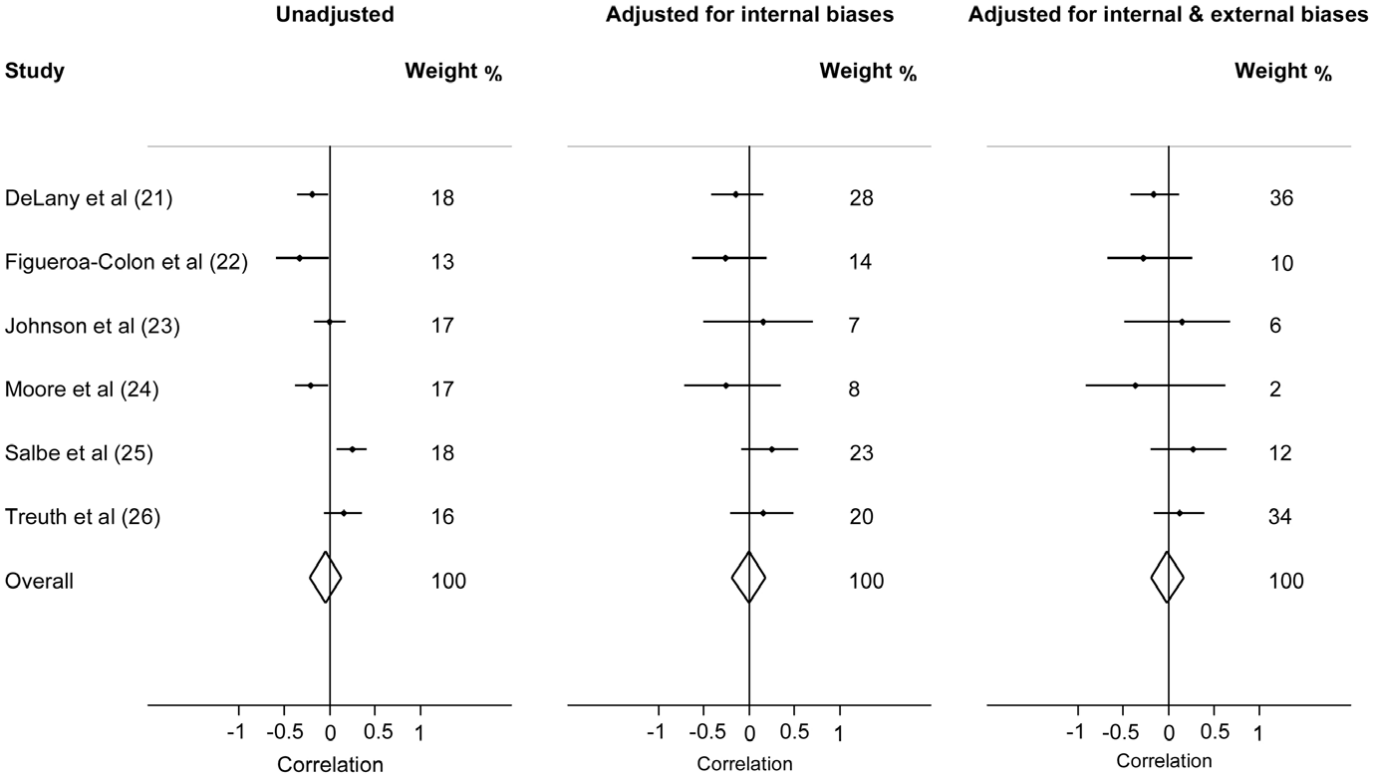
Random-effects meta-analyses unadjusted, adjusted for internal biases and adjusted for internal and external biases. The six studies evaluate the prospective associations between measured physical activity and subsequent change in adiposity in children. The correlation in each source study and the combined correlation are presented, with 95% confidence intervals.

Figure 3 shows the estimated correlations between PA and change in adiposity unadjusted, adjusted for internal biases and adjusted for both internal and external biases, for each study and combined across studies using random-effects meta-analysis. The estimated correlation from the meta-analysis of the unadjusted study results was −0.04 (95%CI: −0.22, 0.14), although there was considerable heterogeneity (I^2^ = 78%). After adjustment for internal biases that reflect lack in study quality, the pooled correlation was 0.00 (95%CI: −0.18, 0.19) and the amount of between-study heterogeneity decreased (I^2^ = 15%). The confidence intervals for each study were wider, due to the uncertainty about how the biases would influence the results. The relative weight given to each study in the meta-analysis changed; the studies increasing in relative weight tended to have biases of more certain magnitude [21], [26] while the studies decreasing in relative weight tended to have more uncertain biases [23], [24].

After adjustment for both internal and external biases, which allows drawing conclusions that are specific to a particular target setting, the pooled correlation between PA energy expenditure and subsequent change in percent body fat was −0.01 (95%CI: −0.18, 0.16), and the studies were apparently compatible (I^2^ = 0%). The relative weight of two studies further increased [21], [26]. The other four studies decreased in relative weight, mainly because either the exposure and outcome measures [22]–[24] or the study population [25] differed considerably to the target setting (Table 1) and there was uncertainty in judging these biases.

To help with the interpretation of this overall pooled correlation, we converted it back to a regression coefficient using the standard deviations given in the paper by DeLany et al [21], i.e. 5.6% for the change in percent body fat and 0.98 MJ/d for PA energy expenditure. The estimated bias-adjusted regression coefficient was −0.05 (95%CI: −1.00, 0.91) change in percent body fat, that is for every 1 MJ/d (239 kcal/d) increase in PA energy expenditure body fat decreased by 0.05%.

## Discussion

This meta-analysis with bias-adjustment allowed a quantitative evaluation of the evidence for the prospective relationship between PA energy expenditure and the change in percent body fat in children. Previous narrative reviews reported that measured PA is not strongly related to the subsequent change in adiposity in children and concluded that PA is not the main determinant of unhealthy weight gain [8], [27]. However, because of the heterogeneous nature of the relevant studies and the difficulties in comparing results presented in different ways, the evidence has not been synthesized quantitatively. This limits the possibility of drawing an overall conclusion and making policy related decisions.

Our analysis provides an overall quantitative synthesis of the evidence-base for decision-makers. The unadjusted results from the six studies gave a combined correlation of baseline PA with change in adiposity of −0.04 (95%CI: −0.22, 0.14), however, the large statistical heterogeneity among studies limits interpretability (I^2^ = 78%). After bias-adjustment the estimates remained similar, but heterogeneity amongst studies had been eliminated (I^2^ = 0%) and the data can now be interpreted with a clearer understanding of the biases. In our view, the observed weak and statistically non-significant bias-adjusted correlation, with a quite tight confidence interval around this null result, provides increased support to policymakers that individual differences in PA may not be a key determinant of unhealthy weight gain in children. It therefore reinforces the idea that regular physical activity may need to be combined with a healthy diet to prevent obesity [34].

After bias-adjustment there was an increase in both the relative weight of studies with biases of more certain magnitude and the width of the confidence intervals for the correlation coefficients of all studies. The width of the confidence interval for the unadjusted pooled result reflects the heterogeneity between studies, while the confidence interval in the adjusted analysis widened due to incorporating the assessors' uncertainty regarding the size of the biases. In other examples, strong evidence of bias in a particular direction may cause both the estimate and confidence interval to change substantially through the bias-adjustment process.

The process of bias-adjustment, at the heart of this method, relies on expert opinion and might be considered to be somewhat subjective. We do not claim that the elicited bias distributions are ‘correct’; we are dealing with epistemic uncertainty, and the distributions express judgments about our beliefs. However, the opinions of several experts are combined so that individual opinions do not unduly influence the final result of the meta-analysis. The experts were chosen for their quantitative and subject-matter skills, and we prefer to incorporate their judgments rather than simply ignore the suspected biases in the studies available. In addition, consistency across studies and transparency is ensured by the very structured and systematic process of bias adjustment. It is conducted in several steps that include for example the identification of internal and external biases, the completion of checklists for each source study, the discussion of each checklist by differing expert groups and the bias elicitation. Although some opinions on biases differed between the assessors, the differences were in general quite small (Figure 1) and mainly related to the width of intervals reflecting different levels of uncertainty about the effect of the biases. Hence, the adjusted estimates for individual assessors were similar to the pooled adjusted estimate (Figure 2).

A similar bias-adjusted meta-analysis has been conducted for a systematic review of dietary energy density and subsequent change in fat mass index in children [35]. This method may be useful and more widely applicable for evidence synthesis across a range of other areas in the population health sciences where studies often cannot be pooled in conventional meta-analyses due to their heterogeneity and differences in design and quality.

In conclusion, this method allowed a quantitative synthesis of the prospective association between PA and fat mass; the result suggests no association. This indicates that objectively measured PA may not be the key determinant of unhealthy weight gain in children, supporting the conclusion drawn in a previous narrative review [27]. The analysis emphasizes the need for higher quality studies presenting adequate analyses.

## Supporting Information

Table S1 **Internal biases identified in the studies.**
(DOCX)
