# Supplementary material for: Objectively Measured Physical Activity and Fat Mass in Children: A Bias-Adjusted Meta-Analysis of Prospective Studies
Source: PLoS One. 2011 Feb 23;6(2):e17205. doi: 10.1371/journal.pone.0017205 (PMC3044163; doi:10.1371/journal.pone.0017205)
Supplement: Table S1 — Internal biases identified in the studies. (DOCX) [file pone.0017205.s001.docx]

**Table S1:** Internal biases identified in the studies.

| **Study** | **Selection** | **Attrition** | **Confounding** | **Exposure/ Outcome** | **Other bias suspected**^1^ |
| --- | --- | --- | --- | --- | --- |
| DeLany  et al [21] |  | - ~15% losses to *FU*. | - No adjustment. |  | - Possible selective reporting (e.g. choice of EE variables). *PB* |
| Figueroa-Colon et al [22] | - - Little information about inclusion and exclusion criteria and the recruitment. | - - ~15% losses *FU*.   - Unclear if there were exclusions from the analysis. | - Use of confounders not justified. - Inappropriate adjustment. |  | - The final linear regression model, derived using stepwise regression, may overestimate the magnitude and significance of the findings. *PB* |
| Johnson  et al [23] | - Few inclusion and exclusion criteria, little information about the recruitment. - No information about *BL* numbers. | - Unclear how many children had fewer *FU*s than anticipated. - Unclear if drop-outs differed from completers. | - Use of confounders not justified. - Unclear whether sex was used as confounder. | - Statistical model inappropriate for our purpose. - The outcome is the ratio of FM and FFM, based on annual measurements. |  |
|  |  |  |  |  |  |
| Moore  et al [24] | - Unclear how many members of the original study were contacted. - 54% of the eligible families participated. - 3% of eligible participants were included in the study; selection unclear. | - 14% of accelerometer data points missing. - 9% of the children had incomplete datasets. | - Use of confounders not justified. - Inappropriate adjustment. | - Categorization of children into PA groups, based on mean accelerometer counts from ages four to 11 years. | - The statistical analysis is not truly prospective since the differences in the mean of annual SSF amongst three activity groups were analyzed. |
|  |  |  |  |  |  |
| Salbe  et al [25] | - Recruitment strategy unclear. - Exclusion of children (22%) due to maternal diabetes. |  | - Use of confounders not justified. |  |  |
| Treuth  et al [26] | - Non-participation rate unclear. | - 13% losses to *FU*. - Number of missing DXA measurements at *FU* 1 unclear. | - Use of confounders not justified. - Inappropriate adjustment. - Self-assessed Tanner stage. |  | - We expect the correlation based on *P* = 0.14 and n = 88 to be overestimated (see Table 2). |
| *BL* = baseline; EE = energy expenditure; *FU* = follow-up; FM = fat mass; FFM = fat free mass; SSF = sum of skinfolds; DXA = dual-energy X-ray absorptiometry assessment; *PB* = proportional bias (this bias was considered on the proportional correlation scale). ^1^All biases were considered on the additive correlation scale unless indicated otherwise. | | | | | |
